# The analysis for time of referral to a medical center among patients with diabetic foot infection

**DOI:** 10.1186/s12875-020-01363-y

**Published:** 2021-01-09

**Authors:** Cheng-Wei Lin, Hui-Mei Yang, Shih-Yuan Hung, I-Wen Chen, Yu-Yao Huang

**Affiliations:** 1 Division of Endocrinology and Metabolism, Chang Gung Memorial Hospital at Linkou, 5, Fusing St., Guishan Dist, 333 Taoyuan City, Taiwan; 2 College of Medicine, Chang Gung University, Taoyuan City, Taiwan; 3 Department of Medical Nutrition Therapy, Chang Gung Memorial Hospital, Taoyuan City, Taiwan

**Keywords:** Diabetic foot infection, Referral time, Lower-extremity amputation

## Abstract

**Background:**

Diabetic foot infection (DFI) is a limb- and life-threatening complication for diabetic patients needing immediate and comprehensive treatment. Early referral of DFI patients to a diabetic foot center is recommended but there appears limited validated evidence, with the association between referral time and clinical outcomes of limb- preservation or in-hospital mortality still lacking.

**Methods:**

This retrospective research studied consecutive type 2 diabetic patients with DFI treated at the major diabetic foot center in Taiwan from 2014 to 2017. Six hundred and sixty-eight patients presented with limb-threatening DFI. After stratifying their referral days into quartiles, the demographic information and clinical outcomes were analyzed.

**Results:**

One hundred and seventy-two patients were placed in the first quartile (Q1) with less than 9 days of referral time; 164 in the second quartile (Q2) with 9-21 days; 167 in the third quartile (Q3) with 21-59 days; and 165 in the fourth quartile (Q4) with >59 days. End-stage renal disease (ESRD), major adverse cardiac events (MACE) and peripheral arterial disease (PAD) were noted as being higher in the Q4 group compared with the Q1 group (25.45% vs 20.35% in ESRD, 47.27% vs 26.16% in MACE and 78.79% vs 52.33% in PAD respectively). The Q1 group had more patients presenting with systemic inflammatory responsive syndrome (SIRS) (29.07% in Q1 vs 25.45% in Q4 respectively, *P*=0.019). Regarding poor outcome (major lower-extremity amputation (LEA) or in-hospital mortality), the Q4 group had 21.21% of patients in this category and the Q1 group had 10.47%. The odds ratio of each increased referral day on poor prognosis was 1.006 with 95% confidence interval 1.003–1.010 (*P*=<0.001). In subgroups, the impact on poor prognosis by day was most obvious in patients with SIRS (OR 1.011, 95% CI 1.004–1.018, *P*=0.003) and those with PAD (OR 1.004, 95% CI 1.001–1.008, *P*=0.028).

**Conclusions:**

The deferred referral of DFI patients to the diabetic foot center might be associated with poor treatment outcome either in major LEA or mortality, particularly in patients with SIRS or PAD. Both physician and patient awareness of disease severity and overcoming the referral barrier is suggested.

**Trial registration:**

Not applicable.

## Main text

### Background

Diabetic foot ulcer (DFU) is the leading cause of non-traumatic lower-extremity amputation (LEA) [1–3]. Early referral of patients with DFU to a specialist multidisciplinary foot team is recommended to prevent limb loss [4, 5]. In real practice, multiple barriers still exist for patients to reach such services despite current service centers being available and appearing to be functioning relatively well [6–9]. When patients experience DFU, diabetic foot infection (DFI) is the leading threatening problem for limb loss and sepsis [10, 11] and is also the most common cause of hospital admissions [1, 3]. Among in-hospital DFU cases, 82% are reported to have DFIs in Europe and 94% in Taiwan [12, 13], and the proportion of DFI is still increasing [14]; nevertheless, little data has discussed the association between the referral time and the treatment outcomes of LEA or in-hospital mortality for patients with DFI. This study intended to elucidate the clinical characteristics and outcome associated with the referral time to a diabetic foot center treating patients with DFI.

## Materials and Methods

### Subjects and DFI treatments

Consecutive type 2 diabetic patients with DFI treated at the major diabetic foot center in Taiwan, the Chang Gung Memorial Hospital at Linkou (a 3,700-bed university hospital), were reviewed from 2014 to 2017 as a retrospective research design. In this center, patients received comprehensive foot care from an interdisciplinary team including diabetologists, cardiologists, plastic surgeons, orthopedists, radiologists, dieticians, and nurse practitioners [6–9]. Six hundred and sixty-eight patients presented with limb-threatening DFI (moderate to severe) according to the definition from Infection Disease Society of American (IDSA) [15] and the International Working Group on the Diabetic Foot (IWGDF) [16] criteria. All patients received comprehensive foot care by a multidisciplinary team [6–9]. Empiric antibiotics were prescribed promptly for these patients initially and subsequently modified according to the results of cultures. Surgical interventions, endovascular treatments, or foot amputations were scheduled after the diabetic foot team reached consensus.

### Data collection

The referral days were counted from foot ulcer development to admission. After stratifying referral days into four categories in quartiles, demographic information was recorded from the patients’ first visit at admission. Medical records included patient age, gender, diabetes duration, HbA1c level, wound size and medical history such as hypertension, retinopathy, neuropathy and history of major adverse cardiac events (MACEs; including coronary artery disease and cerebrovascular accidents). Smokers were classified as currently smoking if they smoked at least one cigarette per day. End-stage renal disease (ESRD) was defined by chronic renal failure with permanent dialysis therapy. Systemic inflammatory response syndrome (SIRS) was defined when matching two or more of the four criteria including abnormal body temperature > 38^o^C or < 36^o^C; tachycardia with pulse > 90 beat per minute; abnormal respiratory rate with > 20 breaths per minute; and abnormal leukocyte > 12,000 or < 4000 /cu mm [15], while peripheral arterial disease (PAD) was defined by non-invasive assessment documentation or symptoms of critical limb ischemia such as cyanosis or gangrene [16].

### Prognosis analysis: LEAs and in‐hospital mortality

Status at discharge was stratified into four groups: non-amputation, minor LEA (i.e., amputation performed including digital amputation or tarsal-metatarsal amputation, as long as it did not involve the ankle area), major LEA (i.e., amputation performed above the ankle joint) or in-hospital mortality. Major LEA and in-hospital mortality were defined as poor prognosis while subjects with limb preservation and minor LEA were used for comparison.

### Data analysis

Comparisons of characteristics between patients with quartiles of referral days were performed using the one-way ANOVA test for continuous variables including age, duration of diabetes, wound size, and HbA1c level, while Pearson’s chi-square test for categorical variables including gender, smoking status, and comorbidities was used. The odds ratios of each increased referral day correlated with adverse outcome (major LEA or in-hospital mortality) among different comorbidities and severity of DFI were calculated and presented in the forest plot. All statistical analyses were performed using the Statistical Package for the Social Sciences (SPSS for Windows, version 19.0, Armonk, NY: IBM Corp.) software.

## Results

### The clinical characteristics among quartiles of referral days in patients with DFI

**Fig. 1:**
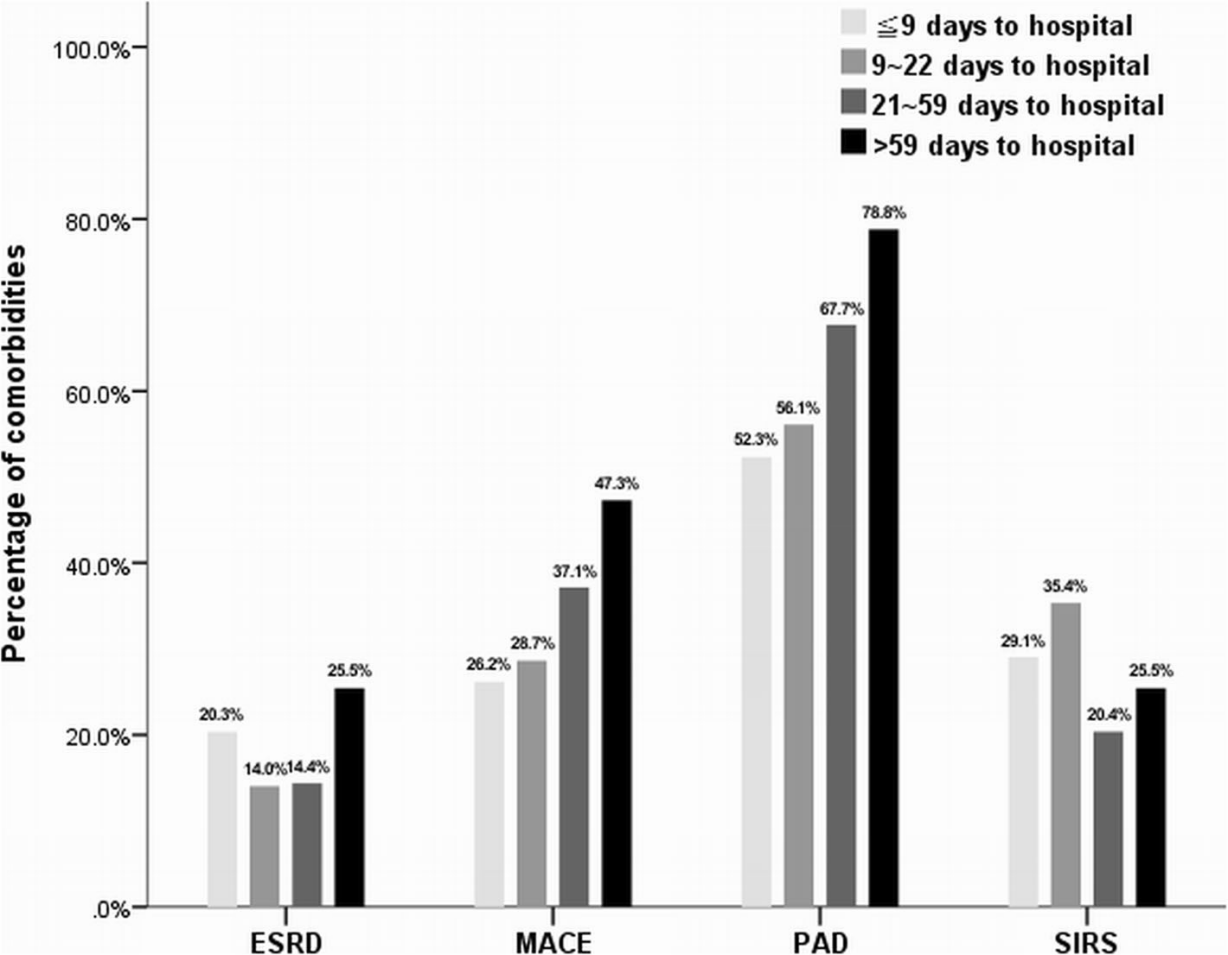
Proportion of comorbidities in quartiles of referral time to hospital The ESRD status, MACE history and presence of PAD were noted as being higher in the latest referral group (fourth quartile) in comparison with the earliest referral group (first quartile) (25.45% vs. 20.35% in ESRD, 47.27% vs. 26.16% in MACE and 78.79% vs. 52.33% in PAD respectively)

The comparison of clinical characteristics of subjects between quartiles of referral days of patients with limb-threatening DFI is shown in Table 1. One hundred and seventy-two patients were enrolled in the first quartile with less than 9 days of referral time; 164 in the second quartile with 9–21 days; 167 in the third quartile with 21–59 days; and 165 in the fourth quartile with > 59 days. The mean age of these patient ranged from 62.96 to 65.19 years without difference and the mean diabetes duration was 12.85 years in the earliest referral group and 15.41 years in the latest referral group but with no significance (*P* = 0.244 in age and *P* = 0.115 in diabetes duration respectively). Male gender predominance was noted in all groups (60–64.02%, *P* = 0.875). The proportions of associated comorbidities were high in patients of all four groups, including hypertension around 70%, retinopathy around 57% and neuropathy around 45%. Of note, the ESRD status, MACE history and presence of PAD were noted as being significantly higher in the latest referral group (fourth quartile, Q4) in comparison with the earliest referral group (first quartile, Q1) (25.45% vs. 20.35% in ESRD, 47.27% vs. 26.16% in MACE and 78.79% vs. 52.33% in PAD respectively) (Table 1; Fig. 1). Regarding symptom presentation, the earliest referral group had more patients presenting with SIRS (29.07% in Q1 vs. 25.45% in Q4 respectively, *P* = 0.019), while HbA1c level was also higher in this group (9.21 in Q1 vs. 8.50 in Q4 respectively, *P* = 0.008).

**Table 1:** Demographics of patients with limb-threatening diabetic foot infection among quartiles of referral time to hospital

	Days from ulcer to hospital (Quartiles)				*P* value
	≤ 9 days (*n* = 172, Q1)	9–21 days (*n* = 164, Q2)	21–59 days (*n* = 167, Q3)	> 59 days (*n* = 165, Q4)	
Age (years)	63.85 (13.52)	62.96 (13.12)	65.62 (13.19)	65.19 (13.63)	0.244
Gender					0.875
Female	63 (36.63%)	59 (35.98%)	64 (38.32%)	66 (40.00%)	
Male	109 (63.37%)	105 (64.02%)	103 (61.68%)	99 (60.00%)	
Diabetes duration (years)	12.85 (10.04)	13.81 (8.90)	14.00 (9.67)	15.41 (10.04)	0.115
Smoking	69 (40.12%)	72 (43.90%)	69 (41.32%)	66 (40.00%)	0.880
Hypertension	126 (73.26%)	105 (64.02%)	117 (70.06%)	117 (70.91%)	0.302
Retinopathy	88 (51.16%)	94 (57.32%)	99 (59.28%)	97 (58.79%)	0.405
Neuropathy	72 (41.86%)	75 (45.73%)	85 (50.90%)	76 (46.06%)	0.423
End-stage renal disease	35 (20.35%)	23 (14.02%)	24 (14.37%)	42 (25.45%)	0.021
Major adverse cardiac event	45 (26.16%)	47 (28.66%)	62 (37.13%)	78 (47.27%)	< 0.001
HbA1c (%)	9.21 (2.61)	9.47 (2.51)	8.84 (2.68)	8.50 (2.55)	0.008
Wound size (cm^2^)	30.05 (58.27)	45.51 (141.00)	21.21 (31.57)	38.74 (74.88)	0.063
SIRS^a^	50 (29.07%)	58 (35.37%)	34 (20.36%)	42 (25.45%)	0.019
Peripheral arterial disease	90 (52.33%)	92 (56.10%)	113 (67.66%)	130 (78.79%)	< 0.001

^a^Systemic inflammatory responsive syndrome

### The association of prognosis and the referral days

**Fig. 2:**
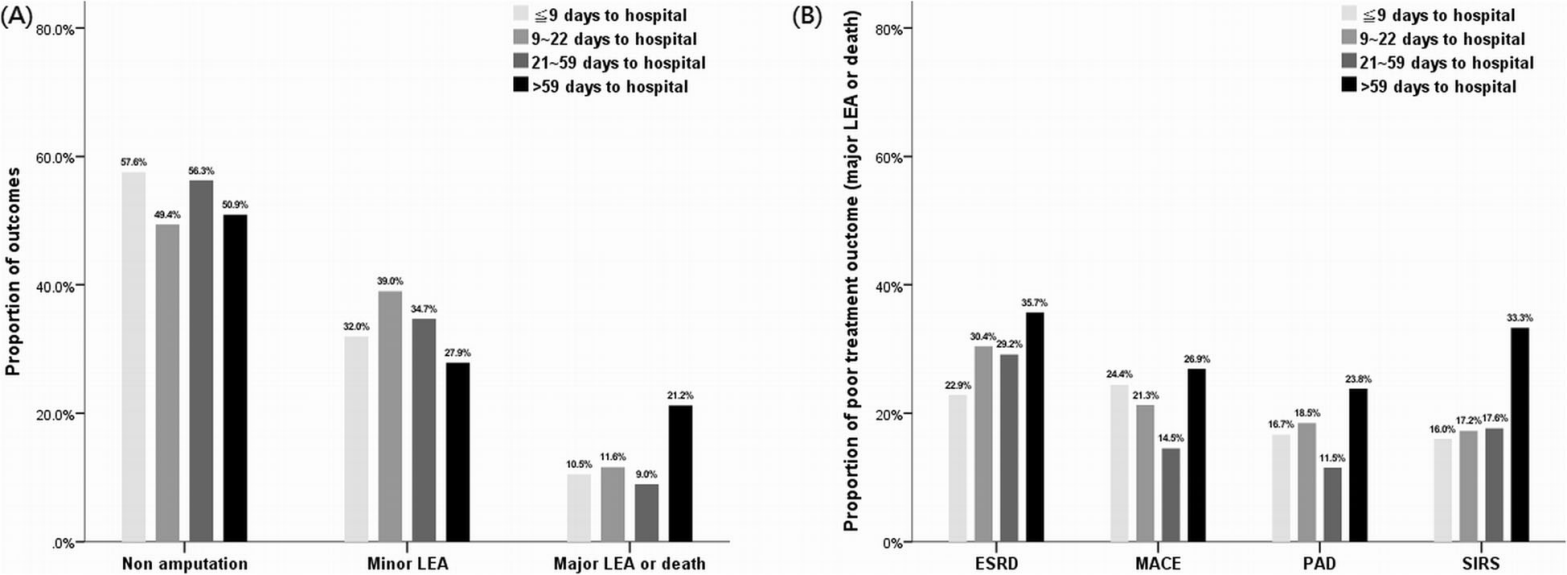
The outcomes among quartiles of referral time to hospital **a** The latest referral group had 21.21% patients resulting in poor outcome (15.15% major LEA and 6.06% in-hospital mortality); and the earliest referral group had 10.47% with poor outcome (8.14% major LEA and 2.33% in-hospital mortality). **b** For the proportions of poor outcomes (major LEA or mortality) among patients with comorbidities, the poorest prognosis occurred in the latest referral group regardless of comorbidities

**Fig. 3:**
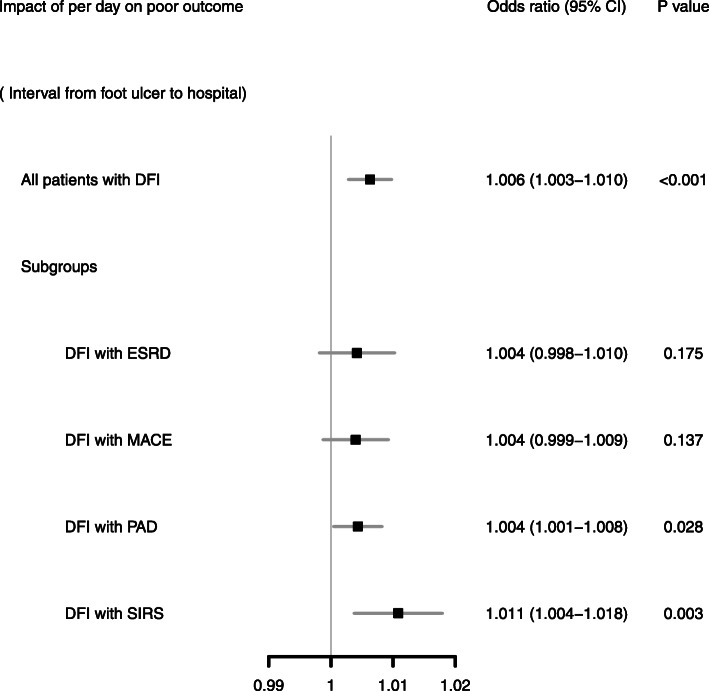
Odds ratios per increased referral day on poor treatment outcome (major-LEA or in-hospital mortality) The odds ratio of each increased referral day on poor prognosis was 1.006 with 95% CI 1.003–1.0010 (*P* < 0.001). The impact was most obvious in patients with SIRS (OR 1.011, 95% CI 1.004–1.018, *P* = 0.003) and those with PAD (OR 1.004, 95% CI 1.001–1.008, *P* = 0.028)

When categorizing the patients into four groups in quartiles of referral days, we found different treatment outcomes (Fig. 2a). For poor treatment outcome, the latest referral group had 21.21% of patients (15.15% major LEA and 6.06% in-hospital mortality) and the earliest referral group had 10.47% (8.14% major LEA and 2.33% in-hospital mortality). When stratifying the patients according to the comorbidities, the poorest prognosis occurred in the latest referral group, though the trend analysis did not show significance (Fig. 2b). Figure 3 reveals the odds ratios of each day (the interval between foot ulcer to the hospital) contributing to the impact on poor prognosis. For all patients, the odds ratio of each increased referral day on poor prognosis was 1.006 (95% confidence interval 1.003–1.010, *P* < 0.001). Regarding the infection severity and comorbidities, the impact on poor prognosis of each increased referral day was most obvious in patients with SIRS (OR 1.011, 95% CI 1.004–1.018, *P* = 0.003) and those with PAD (OR 1.004, 95% CI 1.001–1.008, *P* = 0.028). The impact was non-significantly positive in patients with ESRD (OR 1.004, 95% CI 0.998–1.010, *P* = 0.175) and MACE (OR 1.004, 95% CI 0.999–1.009, *P* = 0.137). Longer referral days among these DFI patients might have associated with poor treatment outcome either major LEA or in-hospital mortality, particularly in subjects presenting with SIRS or coexisting with PAD.

## Discussion

This study investigated the referral time among patients with limb-threatening DFI and still found barriers on early referral to a diabetic foot center for most patients, even under such serious conditions. More than 25% of patients took more than two months to admit themselves to the hospital from disease onset. The study also revealed poor prognosis among patients with longer referral time. Major LEA and in-hospital death were defined as poor treatment outcomes and it was found that the latest referral group (> 59 days) had more patients (21.21%) with poor outcomes (15.15% major LEA and 6.06% in-hospital mortality) and the earliest referral group had 10.47% (8.14% major LEA and 2.33% in-hospital mortality) of such outcomes (Fig. 2a). The referral barrier was also noted in Europe because of various causes including inconsistent or vague guidelines on management of DFUs, and possible unawareness of the risk among patients and general practitioners [4, 5]. In the Eurodiale study, duration of ulcer < 1 week was noted in 17% of patients; 1 week to 3 months in 58%; and > 3 months in 24.9% [4]. Manu et al. investigated the referral times of patients with diabetic foot ulcers across Europe, and reported 13–24% of patients had ≥ one month between the onset of the wound and the diagnosis of DFU, while 11–22% were not referred until one or more months after diagnosis. The other two single-center cohort studies revealed patients with a diabetic foot ulcer had a median time between ulcer onset and diagnosis of 4 (0–247) days (diagnosis delay), and the median time between first review and first referral to the specialist clinic was 15 (0–608) days (referral delay) by Macfarlane and Jeffcoate [17], and 3 days of diagnosis delay with 7 days of referral delay in the study by Antal et al. [18]. In these studies, only Antal et al. investigated the correlation between referral time and clinical outcome and their study suggested healing time was postively correlated to referral time.

This study further disclosed that each increased referral day contributed to the increased poor outcome probability and the tendency was still noted when stratifying the DFI patients according to their comorbidities (Fig. 3). People like these patients with comorbidities are supposed to seek medical help proactively because of their poor health, although surprisingly, in this study, the population coexisting with ESRD, cardiovascular diseases or PAD had a less active attitude to manage their DFI and tended to postpone medical consultations (Fig. 1). Although lacking causal evidence, the reason for the late referral in these patients was supposedly due to the obscureness of the infection symptom by poor perfusion. Both cardiovascular diseases [19, 20] and ESRD [21, 22] are associated with PAD and hence such patients would have poor peripheral perfusion. In theory, poor perfusion is supposed to hinder the symptoms and signs of foot infection by regionalizing the infection only in the foot area, therefore presenting limited inflammation response because of less immune cell reaction [23]. The wound margin tissue necrosis might also develop a barrier to prevent inflammation cytokine dissemination into systemic circulation; consequently, obscure symptoms or signs might lead to unawareness of severity of DFI, particularly in patients with cardiovascular comorbidities. Of note, poorer prognosis was also noted in later referral DFI patients with PAD as well as ESRD or MACE (Fig. 2b). It is known that poor perfusion causes poor tissue oxygen tension and hinders delivery of antibiotics to the infected tissue, thereby influencing poor response to treatment [23]; nevertheless, if the poor perfusion is prolonged in the limb, collateral vessel blood supply would no longer be sufficient to compensate for tissue hypoperfusion, which would then result in increased cell loss and ultimately a local inflammatory reaction [24]. Furthermore, inflammation causes injury in tissue and the damaged cells are broken down and replaced by fibrosis, which presents an additional barrier to oxygen diffusion in the tissue thereby further exacerbating ischemia, resulting in a vicious cycle of poorer perfusion, let alone poor limb outcome.

Unlike the comorbidities, the proportions of SIRS presentation were subtly higher in early referral groups but with limited diversity between the different range of referral days (Fig. 1). The presentation of SIRS in patients with DFI always results from more damaged and inflammatory tissue like necrotizing fasciitis, osteomyelitis or abscess formation [9]. SIRS indicates inflammation beyond the infected site traversing throughout the entire body. Such severe infection suggests higher risks to limb loss or mortality, and will almost certainly turn aggressive for DFI treatment once patients experience obvious symptoms such as SIRS. In our study, the similar proportions of SIRS presentation in four groups meant not all DFI patients developed SIRS as soon as disease onset and those with underlying cardiovascular diseases or ESRD might only develop SIRS when infection is advanced. Given previous research, DFI patients would have poor prognosis once they developed SIRS [9]. Our study further documented that despite the same SIRS presentation on arrival, later referral patients still suffered from poorer treatment outcomes. Accordingly, patients and clinicians should recognize the severity of DFI in a timelier manner and patients should be referred to the hospital with a diabetic foot team, even under obscure symptoms.

To grasp time for treatment is important for some diseases to rescue damaged organs. For example, a concept of “time is muscle” was deliberated over by cardiologists regarding the treatment of primary percutaneous coronary intervention for acute ischemic heart disease, aiming to limit the extent of myocardial damage [25, 26], meaning that severity and extent of myocardial ischemic injury resulting from coronary occlusion could be radically altered by timely intervention [26]. A similar thought of “time is limb” was also mentioned in acute limb ischemia needing early recognition of poor perfusion thereby requiring revascularization to prevent limb loss and life-threatening morbidity [27, 28]. Beyond limb ischemia, in this study, early referral for early treatment is also supposedly as important for diabetic patients having foot infection, and the analysis revealed the probability of poor outcome (major LEA or death) vis-à-vis each increased referral day. The time impact was more obvious when the patient presented with SIRS or PAD on arrival; therefore, delayed referral to the hospital until severe inflammation or critical limb ischemia has set in should be avoided to prevent limb loss or mortality.

This study was limited by a single center and retrospective design, so it is uncertain whether the correlation between comorbidities and referral time could be applied to the causal factors of delayed referral. The prognosis analysis was according to in-hospital outcomes, and long-term limb preservation and survival require further prospective study designs to be validated.

## Conclusions

Deferred referral to a diabetic foot center for patients with limb-threatening DFI might be associated with poor treatment outcome of either major LEA or in-hospital mortality, particularly in subjects with SIRS or PAD. The awareness of the disease severity of DFI and overcoming the barrier of referral is important for both patients and clinicians, and even obscure symptoms in DFI should not be neglected.

## Data Availability

The datasets used and analyzed during the current study are not publicly available due to the restriction of data access according to the ethics committee but may be available from the corresponding author on reasonable request and with permission of the responsible ethics committee.

## Abbreviations

DFU: Diabetic foot ulcer
LEA: Lower-extremity amputation
DFI: Diabetic foot infection
IDSA: Infection Disease Society of American
IWGDF: International Working Group on the Diabetic Foot
MACE: Major adverse cardiac event
ESRD: End-stage renal disease
SIRS: Systemic inflammatory response syndrome
PAD: Peripheral arterial disease
OR: Odds ratio
CI: Confidence interval
